# 

**Published:** 1841

**Authors:** 


### TO SUBSCRIBERS.

We have now fulfilled the promise made at the commencement of our labors as editors and publishers of the American Journal of Dental Science, having completed twelve full numbers according to the pledge given on issuing the first number ; and if subscribers will now fulfil their part, and send in the amount of their dues, these, together with the donations already received from a few liberal and high minded men, will afford ample means to defray all expenses ; otherwise, there will be a deficiency resting upon our hands, which we hope those who did subscribe and send in their names to aid us in this enterprise, will have too much honor to suffer us to lose—and too much pride to allow their names to go before the world, as having received the journal, and defrauded its projectors of the small amount due for their subscription.

For the labor bestowed in the accomplishment of our design to establish the journal, we, at the beginning, looked for no pecuniary emolument, nor do we ask it now, having received a higher reward than wealth can bestow, in the commendations we have so often received for our efforts to improve the condition of our science, from the ablest and most distinguished authorities ; as well from gentlemen of the Medical, as of our own profession ; and also from the best medical journals in Europe and in this country.

The first Dental Surgeons of Europe, among whom we are proud to mention the names of Cartwright, Koecker, and Brewster, have, in letters written in the kindest and most friendly manner, expressed their high satisfaction at the efforts we are making to advance, not only in this country, but also in Europe, the interests of the dental art ; and no inconsiderable portion of our subscribers are of the most distinguished dentists of the various cities of Great Britain.

From the dentists of our own country, we feel happy in saying, that we have received many proofs of kindness and good will, and much to encourage the effort we made in the midst of doubt, surrounded as we were by so many disheartening circumstances. Much may have been said against us that has never come to our knowledge; but we have heard of but two individuals in the United States, having pretensions to a reputable standing in the profession, who have spoken lightly or sneeringly of our efforts to raise the standard of professional excellence; and we would, in all friendship and good will say to them, that it is our earnest desire that they may yet feel the importance of the subject, and unite their talents, and do more for the profession than we have been able to do; and that their labors *in* the profession will distinguish them for something more than the amount of fees they receive for their services; and also, that their labors *for* the profession will be beyond what relates to their own individual interest and benefit.

The Journal will in future be conducted under the immediate direction of the American Society of Dental Surgeons: the permanency of which society, we now consider established beyond all doubt. The late convention in Philadelphia, extracts from the proceedings of which will be published in the first quarterly number in September next, gave decided proofs of its permanent character.

---

### OUR SECOND VOLUME.

It is with sincere pleasure that we announce to our subscribers, that the future destinies of this journal have been entrusted to the fostering care of the American Society of Dental Surgeons. That body has assumed the entire management and control of the work, after the completion of the first volume, on condition that the intentions and engagements of the original publishers shall be fulfilled.

The society has so far modified the name, as to denominate the work hereafter, "The American Journal and Library of Dental Science." It will be issued quarterly, as the former publishers had intended, and the subscription price will be raised, agreeably to their plan, to five dollars per annum.

The propriety of the change of name, results from the fact that one equal half of the work is to be devoted, as heretofore, to the republication of standard works on dental theory and practice; and the necessity of advancing the price to five dollars, arises from the circumstance that no *special subscriptions* are solicited, as heretofore; but the work is made to depend on the funds of the society, aided by ordinary subscription. Three dollars per volume will not defray the expense of a work of this magnitude, having so small a subscription list as the dental profession will be likely to supply.

In order to give value to each copy, only 400 copies will be issued, and the average number of pages of each quarterly number of the journal, will be at least 142; or 568 pages per annum; equal to the 12 numbers of the first volume.

As the society has assumed the entire control of the journal, that body has appointed two editors, C. H. Harris, M. D. of Baltimore, and Solyman Brown, M. D. of New-York, for the year ensuing.

Associated with the editors, are the following twenty collaborators, whose duty it is to procure and furnish matter for the pages of the work, and to aid its circulation—each being *ex officio agents* for receiving subscription and transmitting the moneys to the treasury:

H. H. Hayden, M. D.—*Baltimore.*
Eleazar Parmly, M. D.—*N. York.*
E. Maynard, M. D.—*Washington City.*
E. Townsend,—*Philadelphia.*
Ludolph Parmly, D.D.S.—*Mobile.*
Vernor Cuyler, M. D.—*Hart. Con.*
Lewis Roper, M. D.—*Philadelphia.*
E. Baker, M. D.—*New-York.*
E. Noyes,—*Baltimore.*
Alex. Nelson, M. D.—*Albany.*
Doctor Rogers,—*Cincinnati.*
Horatio N. Fenn, M. D.—*Rochester.*
B. A. Rodrigues, M.D.—*Charleston.*
Hudson S. Burr, M. D.—*Philadel.*
Edward Taylor,—*Natches.*
Leonard Kœcker, M. D.—*London.*
C. S. Brewster,—*Paris, Fr.*
I. I. Greenwood,—*New-York.*
H. M. Hayden,—*Baltimore.*
G. G. Brewster,—*Portsmouth, N. H.*

Inasmuch as the editors have already received many kind proffers of assistance in the department of agencies for the journal, they take the liberty of making the following appointments, and of saying to the profession at large, that the Journal will be regularly forwarded as directed, to all subscribers who shall have remitted the amount of subscription to either of the following agents:

S. Highley, *Bookseller, London.*
E. Gidney, *Dentist, Manchester,*
J. N. Frink,—*Portland, Me.*
Bradbury & Soden,—10 *School-st. Boston.*
E. J. Dunning,—*Buffalo.*
Benj. Strictland,—*Cleveland, O.*
Nicholas Clute,—*Louisville, Ky.*
B. B. Brown, M. D.—*St. Louis. Mo.*
Geo. W. Parmly,—*N. Orleans.*
J. Harris, M. D.—*Georgetown, Ky.*
O. P. Laird, M. D.—*Columbus, Ga.*
W. R. Scott,—*Raleigh, N. C.*
F. B. Chewning, —*Richmond, Va.*

All agents are respectfully notified that the Editors make themselves responsible if they supply any subscriber before the amount of his subscription has arrived at the Treasury of the Society, kept by E. Baker, M. D. No. 6 Warren street, New-York.

---

### POPULAR ESSAYS,

### ON IMPORTANT SUBJECTS IN DENTAL SCIENCE.

The profession are respectfully informed that five of the thirteen Essays, ordered by the American Society of Dental Surgeons, are now ready for distribution, at one dollar and fifty cents per hundred.

These Essays are stereotyped as fast as the writers prepare them; and thus every number can be published from time to time, as the orders from various parts of the country may demand.

Orders containing cash remittances should be forwarced to the Recording Secretary of the Society, Solyman Brown, M. D., No. 13 Park Place, New-York, who will transmit the Essays by such conveyance as shall be prescribed. The postage is too much to allow of their being conveyed by mail. As they are not periodicals, letter-postage will be demanded. Private conveyance must therefore be employed. The five Essays now ready, are the following:—

On the Utility of Artificial Teeth—by Chapin A. Harris, M. D., Baltimore.

On the Tooth Ache and its Cure—by Vernor Cuyler, M. D., Hartford.

On the Ulceration of the Fangs of the Teeth, and the Best Methods of Cure—by Elisha Baker, M. D., New-York.

On the Preservation of the First Set of Teeth—by Enoch Noyes, Baltimore,

On the Importance of Regulating the Teeth of Children during the Progress of the Second Dentition—by Solyman Brown, M. D., New-York.

The object of the society in publishing these Essays in the form of cheap tracts, is to enable every individual in the profession to do something if he choose, to inform community of their real wants on the subject of dental practice.

A few dollars annually expended for this object, by every respectable dentist in the United States, would do much in opening the eyes of the public to the urgent importance of judicious dental practice, in every family in the land.

When this system of Essays shall be complete, some one or more of them will be adapted to the wants of every individual in society; and the dentist who feels the importance of disseminating correct information, for the benefit of the public, as well as for the benefit of his profession, will not, we think, allow himself to neglect so favourable an opportunity of doing good.

The remaining essays are as follow:—

On the Importance of Stopping Carious Teeth—by J. H. Foster, M. D New-York.

On the dangerous effects of salivary Calculus—By Edward Maynard, M. D. Washington.

On the best method of preserving the natural teeth—By L. S. Parmly, M. D. New Orleans.

On the necessity of extracting diseased Teeth—By E. Townsend, Philad.

On the diseases of the Gums—By H. H. Hayden, M. D. Baltimore.

On the First Dentition—By C. A. Harris, M. D. Baltimore.

The acting members of the Society, by which are meant those who have paid their annual dues of five dollars to the treasurer, are entitled by a resolution of the society passed at its last meeting, to twenty-five copies of each of the Essays without charge. Orders for the same will meet the punctual attention of the Recording Secretary.

NOTICE.—The Diplomas of the American Society of Dental Surgeons, are now ready for delivery to the members. A Certificate from the Treasurer, E. Baker, M. D., No. 6 Warren-st. N. York, presented to the Recording Secretary, Solyman Brown, M. D. 13 Park Place, N. Y. and stating that the sum of ten dollars in addition to the annual dues of five dollars has been sent to the treasury, will authorize the secretary to issue the Diploma.

---

A Catalogue of the Officers and Members of the American Society of Dental Surgeons, for 1841—2, has been published by order of the Society, copies of which on parchment, satin, or fine drawing or silk paper, can be had at one dollar each by applying to the subscriber. The satin, or silk paper copies may be sent by mail, without great expense or injury.

SOLYMAN BROWN, Recording Secretary.

### INDEX.

A Page

Abuse of Dental Practice, thoughts on, by John C. McCabe, Dentist. 133
Abuses of Dentistry, vol. 1. No. 1. . 9
Account of a remarkable tooth, by E. Baker, vol. 1. No. 1. . . . 14
Aching tooth, by John N. McJilton, 77
Address of Pub. Com. vol. 1. No. 1, 3
Advice on the teeth, by C. S. Brewster, . . . . . . . . 125
Advancement of Med. Science Warsaw University, . . . . 240
Alveolar abcesses, case of, by Augustus Woodruff Brown, . . 55
American Jour. of Med. Sciences 122
" Society of Dental Surgeons 177
" " " " information concerning, by Solyman Brown, M. D. . . . 190
Anatomy of the teeth of man and other animals, by Pro. Blandin, translated from the French, with notes by H. Burdell, M. D. vol. 1. No. 2. . , . . . . . . 13
" of the human teeth, vol. 1. No. 2. . . . . . . 20
Anatomical discovery, the ligamentum dentis, by F. H. Flagg vol. 1 No. 1. . . . . . 18
An Essay on artificial teeth, by Leonard Koecker M. D notice of 180
Annual circular of the Washington University of Balt, Med. depot 239
Artificial teeth, pivoting method of inserting. . . . . . 125
Asphyxia, or appearance of the teeth, of those who have died from strangulation, by H. H. Hayden M. D. . . . . 212

B

Baker E. account of a remarkable tooth, &c. vol. No. 1 14
,, on the treament of the nerve of the tooth, &c. . . . 17
Baltimore College of Dental Surgery. . . . . . . .120
Bibliography, the select Medical Library & Eclectic Jour. of Med. 121

Page

Bibliography, Maryland Med. and Surg. Jour. . . . . 124
" " American Jour. of Med. Sciences. . . . . . 122
" " Dental Practice. . 123
" " An Essay on artificial teeth by Leonard Koecker M. D. &c. &c. . . . . 180
Bibliography, Traité des dents, by C. A. Jamet. . . . . 243
" Researches on the development &c. of the Teeth, by Alexander Nesmyth.
Brewster C. S. Advice on the teeth. . . . . . 125
Brockway Josephus, on Galvanic action of Metals in the mouth. 172
Brown Solyman A. M. review of the Dental Art, a practical treatise on Dental Surgery by Chapin A. Harris M. D. vol. 1. No. 1 8
" " Remarks on professional morality. vol. 1. No. 2 1
An extraordinary instance of the force of hereditary principle, &c. vol 1, No. 1, . . . . 15
Review, vol. 1, No. 2. . . . 19
On the Osseous Union of the Teeth. . . . . . 89
Information concerning the Am. Soc. of Dental Surgeons, 190
On the cure of Tooth Ache, 227
Use of As[illegible]estos in stopping a certain class of carious teeth. 241
Notice of Bond's Valedictory, 257
Popular Essays on important subjects in Dental Science . 280
Our second Volume . . 179
Notices . . . . . 290
Bryan E, Memoirs of the Life of the late Mr. John Greenwood, Mechanical & Surgical Dentist, 73
" " " "73 113
Burdell Harvey, M. D. Translation from Blandin's Dental Anatomy vol. No. 2 . . . 13
" " " 66—126

Page
Bond, Valedictory address of, before the Baltimore College of Dental Surgery 241
C
Caries of the Teeth, . . . 79
Catalogue of Dental Works placed at the disposal of the Publishing Com. vol. No. 1 . . . 23
Cleanliness of the Teeth, &c. by C.A.Harris, M. D. vol.1. No.2 9
Clark A. Letter from . . 93
College of Dental Surgery, Balt. 120
" Hampden Sidney, Medical department, . . . 239
Communication on the impropriety of filling teeth with two kinds of metals, by L. Mackall, M. D. 86
Constitutional disease, effects of, on the health and structure of the human teeth, by James D. McCabe, Surgeon Dentist. 60
Convention Dentists,'doings of, vol. 1, No. 2 17
Correspondents . . . 184
Crane Doctor's letter . . 219
Correction. . . . . 246
Cure of Tooth-ache, by Solyman Brown, M. D. . . 227
D
Delebarre C. F. Extract from his treatise on 2d dentition, translated by C. A. Harris, M. D. vol.1 No. 1. . . . . . 10
Dental Surgery, a practical treatise on, by C. A. Harris, M. D., review of, by S. Brown, vol. 1. No.1 8
" " Balt. College of 120
" " Observations on the qualifications necessary to a practitioner of, &c. by C. A. Harris, M. D. . . 100
" Surgeons, the Amer. Soc. 177
" " . . . . 242
" " information concerning the Amer. Soc. of by Solyman Brown, M. D. 190
" Surgery, Introductory lecture delivered before the class of the Balt. College of, by C. A. Harris, M. D. D. D. S. 198
Dental Library, vol 1, No 1 17
" Practice, thoughts on the abuse of, by J. C. McCabe, Surgeon Dentist, 133
" " " " 123
" Science, historical sketch of the progress of, vol 1 No 2 14

Page
Dental Science, for the Am. Jour. of, by E. Noyes 129
" " " 172—176
Dentistry, abuses of, vol 1 No 1 9
Dentist's Society, National 157
" Convention, doings of, vol. 1 No 2 . . . . . 17
Dentologia . . . 120
" a poem, review of, by C.A. Hartis, M. D. 69
Dentaire, Anatomie du System, Prof. Blandin's, extracts from, trans. by H. Burdell, vol 1 No 2 13
" " " 66—126
Dents, Traité des &c. notice of 243
Dentition, Premature, by S. Brown vol 1 No 2 12
" Second, extract from a treatise on, by C. F. Delabarre, translated by C. A. Harris, M. D. vol 1 No 1 10
Development and structure of the Teeth 228
Discovery, anatomical, the ligamentum dentis, by F. H. Flagg, vol 1 No 1 18
Dissector, London. 78
Diseases of the Human teeth and bones, application of the doctrine of septic fluids to explain some of which, by Dr. Mitchell 79
Doctor Crane's Letter 219
E
Effects of constitutional disease on the health and structure of the human teeth, by J. D. McCabe; Surgeon Dentist 60
Exostosis of the upper jaw, by B. A. Rodrigues, M. D. 88
Extracts from a treatise on second dentition, by C. F. Delabarre, M. D. translated from the French, by C. A. Harris, M. D. vol 1 No 1 10
Extracts from Professor Blandin's dental anatomy, translated from the French, by Harvey Burdell, M. D. vol 1 No 2 13—66—126
Extracts from the minutes of the dentists' convention 157
Extract of a letter from Dr. Harris to the New York Editor, 217
F
Facialis, Neuralgia, by D. S. Grandin, M. D. 214
First period of the progress of dentistry in Egypt, China & Greece to the time of Aristotle, vol 1 No 2 14

Page
Fourth period 66
Fifth " 67

G

Galvanic action of metals in the mouth, by Josephus Brockway 172
German remedy for tooth-ache, old 226
Gidney, E. Esq. catalogue of dental works placed by him with Pub. Com. vol 1 No 2 23
Greenwood John, memoirs of the late Mr., mechanical & surgical dentist, comp. by E. Bryan 73, 97, 113
Grandin, S., M. D. on Neuralgia Facialis 214
Growth & formation of the human teeth, an investigation on the nature of, &c. by H. H. Hayden, Esq. 137

H

Hamden Sidney College, Med. Dep 239
Hill, A. Letter from . . 287
Harris, C. A. M. D., cleanliness of the teeth, its importance to their health and preservation, vol 1 No 2. . . 9
" Introductory lecture 198
" observations on the qualifications necessary to a practitioner of dental surgery, &c. 49
" " translation from C. F. Delabarre's treatise on second dentition, vol 1 No 1 10
" " Review of Dentologia, a poem, by S. Brown, A. M. 68
" the Dental Art, a practical treatise on dental surgery, reviewed by S. Brown, A. M. vol 1 No 1 8
" " Ex. of a letter from to the New York Editor 217
Hayden Horace H. Esq. an investigation on the nature, growth & formation of the human teeth &c. 137
" " Asphyxia, or the appearances of the teeth of those who have died from strangulation 212
Hayden, observations on the use and functions of the salivary Lachrymal, and other glands of the human system 262
Historical sketch of the progress of dental Science, translated from the French by H. Burdell, M. D. 66
Hullihen, S. P. observations on tooth-ache 105
Human teeth, effects of constitutional disease on the, by James D. McCabe, Surgeon Dentist. 60

Page

I

Important announcement 125
Information concerning the Amer. Society of Dental Surgeons, by Solyman Brown, M. D. 190
Inserting artificial teeth, pivoting method 125

J

Journal of Medical Science, the American - - - 122
" Dental Science, the American, prospectus of vol 1 No. 1 5
" " " vol. 1. No. 2. 13
" The Maryland Medical and Surgical 124
" Of Dental Science, for the American, by Enoch Noyes 129
" " Approval of by Ameri. Society of Dental Surgeons 190

K

Koecker Leonard, M. D. his method of Treating the Lining Membrane of a tooth when exposed 135
" " An Essay on Artificial Teeth, notice of 180

L

Lecture Introductory, delivered before the class of the Baltimore College of Dental Surgery by Chapin A. Harris, M. D. D. D. S. 198
Letter to Drs. Harris and Parmly from Enoch Noyes vo. 1 No. 2 18
" from Mr. A. Clark, 93
" " Enoch Noyes in reply to Mr. A. Clark 129
" " W. W. H. Thackston in reply to Mr. Shepherd on the use of the Turn-Key 185
Letter, extract of a, from Dr. Harris to N. Y Editor 217
" Dr. Crane's 219
" from G. Merryman on osseous union of the teeth 176
" from John C. Mc'Cabe to the Baltimore Editor 225
Library, Dental, vol. 1 No. 1 17
Ligamentum Dentis, vol. 1 No. 1 18
Life of the Late Mr. John Greenwood, Mechanical and Surgeon Dentist, Memoirs of, compiled by E. Bryan. 113
London dissector 78

M

Mackall L., M. D. Communication on the impropriety of plugging teeth with two kinds of metal in the same mouth 86

Page

Maryland Medical and Surgical Journal,notice of vol 1 No 2—24, 124
McCabe James D. Surgeon Dentist on the effect of constitutional disease on the health and structure of the human teeth 60
" John C. on the abuse of dental practice 133
" " letter from to Baltimore Editor 225
Medical Library the select and Eclectic Journal of medicine 121
" Science the Amer. Journal of 122
" and Surgical Journal, Maryland 124
Memoirs of the Life of the Late Mr John Greenwood Mechanical and Surgeon Dentist, compiled by E Bryan 73—97—113
Memoranda from the note book of B A Rodrigues 175
Merryman G. on the osseous union of the teeth 176
Mitchel Dr. Samuel. application of the doctrine of the septic fluid to explain some of the diseases of the human teeth and bones 79
Morality Professional, by Solyman Brown A. M. 1

N

National Dentists' Society 157
Nerves of the teeth, on the treatment of the, by E Baker 170
Nerves living, Destruction of &c. &c. by Solyman Brown M. D. 227
Notice 196
" of Stockton's Establishment 248
Notices, Miscellaneous 239
Noyes Enoch, letter from, vol 1 No 2 18
" " " " 129
Nuralgia Facialis, by S. Grandin 214

O

Observations on the quallifications necessary to a practitioner of dental surgery &c &c by Chapin A. Harris M. D. 49
Odontalgia by S. E. Hullihen 105
Old German remedy for tooth-ache 226
Osseous union of the teeth, remarkable cure of by Eleazar Parmly vol 1 No 1 17
" " Solyman Brown 87
" " G. Merryman 176
Our Next Volume 197
" Subscription 244

P

Patrons, to our 128

Page

Parmly Eleazar, on osseous union of the teeth vol 1 No 1 17
Plugging teeth with two kinds of metal in the same mouth, communication on the impropriety of, by L Mackall M, D 86
Plugging teeth by S L Stringfellow 111
Practitioner of Dental Surgery, observations on the qualifications necessary to, &c by Chapin A Harris *M* D 49
Practice Dental, thoughts an the abuse of, by John C McCabe 133
Professional Morality, remarks on by S. Brown A M vol 1 No. 2 1
" bretheren, address of the Pub. Com. to their, vol 1 No 1 3
Prospectus of Amer. Jour. Dent. Science vol 1 No. 1 5
Publishing Committee, Notice of 112

Q

Qualifications necessary to a practitioner, observations on the, by Chappin A Harris *M* D 49

R

Remarks on professional morality by Solyman Brown A. M. vol 1 No. 2 1
Remarkable tooth, account of by E Baker vol 1 No. 1 14
" " case of osseous union of the teeth by Eleazar Parmly vol 1 No. 1 17
" Alveolar Abscess &c &c by Agustus Woodruff Brown 58
Remedy for the tooth ache, Old German 226
Reprint valuable 247
Review of the Dental art a practical treatise on Dental Surgery by Chapin A Harris M D. by S. Brown A M vol 1 No 1 8
" of Dentologia a Poem by S. Brown A M, by Chapin A. Harris *M* D 69
Rodrigues B A., M D exostosis of the upper jaw 88
" Memoranda from the note book of 175
" an Essay on the mode of treating caries of the teeth to which is addeed a general view of the Anatomy and Physiology of the Teeth . . . 282

S

Science, Dental, of the progress of, 66
Second period, of the progress of dentistry vol 1 No. 2 15

Page
Shepherd S M on the use of the Turn-Key 59
Society, National Dentist 157
" the American of Dental Surgeons, to . . . 177
" " " constitution of 159
" " " By-Laws 164
" " " Members elected to it 168—9
" " " Appointment of to specified duties 168—9
Stockton's Establishment for teeth &c &c 248
Stringfellow S L on plugging teeth 111
Subscribers, notice to, 128—179—224
" names 94
Supcription, our 244
" from the several states 245
Surgery, Dental, observations on the qualifications necessary to by Chapin A Harris M D 49
" " Baltimore College of 120
" " Introductory lecture delivered before the class of the Balt.College of, by Chapin A Harris M D D D S 198

T

Teeth, an investigation on the nature of the growth,and formation of the human &c &c by H H Hayden Esq'r. 137
" Anatomy of men and other animals translated from Blandin's dental anatomy, by Harvey Burdell M D vol 1 No. 2, 126
" Osseous union of, remarkable case by Eleazar Parmly vol 1 No. 1 17
" Cleanliness of the,its importance to their health and preservation by C. A Harris M D vol 1 No. 2 9
Teeth, Human, effect of constitutional disease on the health and structure of the, by James McCabe Surgeon Dentist 60
" A Poem on the diseases of the, by Solyman Brown A M 69
" Caries of the 79
" and bones, application of the doctrine of the septic fluids to explain some of the diseases of the of human, by Dr S L Mitchel 79
" plugging, communication on the impropriety of,with two kinds of metal in the same mouth by LeonardMackall M D 86

Page
Teeth, of the osseous union of, by Solyman Brown 89
" plugging, by S L Stringfellow 111
" A word of advice on the, by C S Brewster 125
" Artificial, pivoting method of inserting 125
" development of, in aged persons 126
" on the treatment of the nerves of the, by E Baker 170
" on the osseous union of the teeth, by G. Merryman 176
" notice of Leonard Koecker's M D on artificial 180
" asphyxia, or the appearance of the, of those who have died from strangulation by H H Hayden M D 212
" on the development and structure of the 228
" on the use of asbestos in stopping certain kinds of carious by Solyman Brown M D 241
" Stocktons Establishment for, &c 248
Tooth, with drawing, account of a remarkable by E Baker vol 1 No. 1 14
" Aching 77
" ache, observations on, by S. P. Hullihen 105
" ache from exposure of the nerves 106
" " fungus of the nerve 107
" " the confinement of pus in the internal cavity 108
" a diseased state of the periosteum covering the fang 109
Tooth-ache from sympathy 110
" Dr Koecker's method of treating the lining membrane of the, when exposed 135
" ache cure of &c by Solyman Brown M. D. 227
" an old German remedy for 226
Translation from La Lancette by J J Greenwood 258

U

Use of the Turn-Key, by S M Shepherd 59

V

Volume, our next 197

W

Washington University of Baltimore Med. Dept. annual circular 239
Warsaw University 240

SOLYMAN BROWN.

---

The collaborators of this Publication, in addition to the Editors and Publishing Committee, are,—

H. H. Hayden, M. D Baltimore
J. F. Flagg, M. D Boston.
B. A. Rodriguez, M. D. Charles S. C.
C. S. Brewster, Paris, France.
Leonard Koecker, M. D. London, Eng.
Enoch Noyes, Baltimore,
Harvey Burdell, M. D New York.
J. P. Hullihen, M. D. Wheeling, Va.
Leonard Macall, M. D., Baltimore.
E. Maynard, M. D., Washington City.
James D. McCabe, Richmond Va.
S. M. Shepherd, Petersburg, Va.
George Merryman, Baltimore.
.S Grandin, M. D., New-York.
W. W. H. Thackston, Va.

---

### NOTICE TO CONTRIBUTORS.

Several articles have been postponed for want of room. They will appear in the second volume.

Besipes these, the Baltimore Editor had prepared several pages of Bibliographical Notices, which are reluctantly reserved for a future number. Hereafter the editors intend to give early information of all new publications in Europe or America, relating to the dental profession.

---

**Cutlery.**--WILLIAM DAILY, Surgical and Dental Instrument Maker, *Sharp Street, four doors South of Market, Baltimore.* All work done at this establishment, will be of the best materials, neatest workmanship, and at the most reasonable prices. Orders filled at the shortest notice. References, Physicians and Dentists generally, of Baltimore.

AGENTS FOR THE

### AMERICAN JOURNAL OF DENTAL SCIENCE.

Doct. W. N. McKenney, *Fredericksburg, Va*
F. B. Chewning, *Richmond, Va.*
A. Pierce, *Sussex Court House, Va.*
Chas. S. Pleasants, *Painesville, Ohio.*
David Walker & Asahel Jones, 174 *Fulton Street, New York.*
Edward Taylor *Maysville K. Y.*
O. P. Laird, M. D., *Columbus, Ga.*
John D. Chevalier, 135 *Fulton-st. N. Y.*
Geo. Washington Parmly, *N. Orleans.*
A. W. Brown, *New-York.*
F. M. Boykin, M. D. *Smithfield, Va.*
G. McDonald, M. D. *Macon, Geo.*
E. Maynard, M. D. *Washington City.*
McAllister & McNaughton, *Albany.*
M. K. Bridges, *Brooklyn.*
Blanding & Avery, *Columbia, S. C.*
Eleazer Gidney, *England.*
S. Wheeler, M. D *Murfreesboro, N. C.*
W. H. H. Davis, M. D. *Cassville, Oneida* Co. *New-York.*
B. O. Rodriguez, M. D. *Charles. S. C.*
Ferguson & Goddard, *Louisville, Ky.*
L. S. Stringfellow, *Baltimore.*
J. P. Norman, *Little Rock, Arkansas.*
E. E. Crowfoot, *Middletown, Conn.*
Samuel Highley, *Fleet-st. London.*

